# Efficient Thorax Disease Classification and Localization Using DCNN and Chest X-ray Images

**DOI:** 10.3390/diagnostics13223462

**Published:** 2023-11-17

**Authors:** Zeeshan Ahmad, Ahmad Kamran Malik, Nafees Qamar, Saif ul Islam

**Affiliations:** 1 Department of Computer Science, COMSATS University Islamabad, Islamabad 45550, Pakistan; zeeshanresearch@outlook.com; 2 School of Health and Behavioral Sciences, Bryant University, Smithfield, RI 02917, USA; nqamar@bryant.edu; 3 Department of Computer Science, Institute of Space Technology, Islamabad 44000, Pakistan

**Keywords:** thorax disease, chest X-ray, Deep Convolutional Neural Network (DCNN), image processing, classification

## Abstract

Thorax disease is a life-threatening disease caused by bacterial infections that occur in the lungs. It could be deadly if not treated at the right time, so early diagnosis of thoracic diseases is vital. The suggested study can assist radiologists in more swiftly diagnosing thorax disorders and in the rapid airport screening of patients with a thorax disease, such as pneumonia. This paper focuses on automatically detecting and localizing thorax disease using chest X-ray images. It provides accurate detection and localization using DenseNet-121 which is foundation of our proposed framework, called Z-Net. The proposed framework utilizes the weighted cross-entropy loss function (W-CEL) that manages class imbalance issue in the ChestX-ray14 dataset, which helped in achieving the highest performance as compared to the previous models. The 112,120 images contained in the ChestX-ray14 dataset (60,412 images are normal, and the rest contain thorax diseases) were preprocessed and then trained for classification and localization. This work uses computer-aided diagnosis (CAD) system that supports development of highly accurate and precise computer-aided systems. We aim to develop a CAD system using a deep learning approach. Our quantitative results show high AUC scores in comparison with the latest research works. The proposed approach achieved the highest mean AUC score of 85.8%. This is the highest accuracy documented in the literature for any related model.

## 1. Introduction

Thoracic illnesses are widespread and significant health issues that affect a lot of individuals all over the globe. For instance, pneumonia kills roughly 4 million people annually and infects around 450 million worldwide (seven percent of the total population). One of the most used forms of radiological examination for identifying thoracic disorders is chest radiography, often known as chest X-ray (CXR) [1,2]. Globally, countless chest radiographs are produced each year, and virtually all of them are examined visually by humans. This is much expensive, time consuming, operator biased, and not able to take advantage of precious large data. It also needs a high level of skill and focus [3]. A significant public health issue in many nations is the absence of qualified radiologists who can interpret chest radiographs. Therefore, it is essential to create an automated technique for thoracic illness detection on chest radiographs using computer-aided diagnosis (CAD). The ChestX-ray14 dataset was provided by Wang et al. [1,4,5], who recognized its value and used it to assess automated techniques for diagnosing 14 thoracic illnesses using chest radiography.

For diagnosis using radiology, chest X-ray is currently the most used test in hospitals. The intricate pathological anatomy of many diseases and accompanying lesion regions make the automated chest X-ray classification a tough assignment [6]. In hospitals, chest X-ray analysis is entirely at the discretion of a radiology professional who may diagnose the disease and the portion of the body afflicted by the lesion. To appropriately categorize a range of illnesses by analyzing chest X-ray images, computer-aided diagnosis is critical [7,8,9]. This may be achieved with the help of a CAD that arises from the laborious task of converting human knowledge into artificial intelligence’s language [10,11,12].

For decades, radiography has been an essential tool for detecting medical disorders and helping in therapy administration [1,10]. The growth of the medical business has resulted in the employment of automatic categorization techniques based on machine learning paradigms; nevertheless, the data are meaningless in the absence of a professional diagnostician [13]. Dealing with radiological X-ray data necessitates extensive experience and knowledge. Because of the gravity of the situation, an expert will probably need a significant amount of time to review the X-ray data. Even now, there is a chance that weary paramedics may make an error that might have been prevented [14]. Similarities between some diseases, such as pneumonia, which overlaps with various ailments, have exacerbated the problem [13]. As a result, there is a demand for radiological X-ray data automation that can categorize diseases in ways that the human eye or expert knowledge cannot. According to the World Health Organization (WHO), more than 66% population of the world lacks access to modern radiology diagnostic and specialist skills [15]. Atelectasis, effusion, cardiomegaly, masses, infiltration, emphysema, pneumonia, consolidation, pneumothorax, fibrosis, nodules, pleural thickening, edema, and hernia are some of the fundamental thoracic illnesses that may be detected using a chest X-ray. Additional thoracic (CXR) research can be seen in  [16,17,18]. Issues surrounding the detection and treatment of illness are growing progressively critical due to the COVID-19 epidemic. Researchers may now conduct their research using CXR for free on numerous digital platforms. This publicly accessible dataset contributes to bioinformatics and computer science by providing readers with an overview of the findings described in these reports [19]. Several pre-existing methodologies and procedures have enabled the utilization of these massive CXR recordings [3,20,21].

In this work, it is important to utilize techniques for deep feature extraction to achieve the best classification of 14 thoracic diseases. The major contributions of the paper are provided below:

- To identify the state-of-the-art technique for thorax disease classification and localization;
- To develop and design an architecture for thorax diseases, multi-classification, and their localization and implement it through the proposed model;
- To evaluate the proposed model and achieve higher accuracy (AUC-ROC) as compared to the state-of-the-art research.

The remainder of the paper is organized as follows. Section 2 discusses the related works. Section 3 describes the ChestX-ray14 dataset, its preprocessing, and related issues. Section 4 explains the proposed Z-NET model details. Section 5 describes preparation of a dataset for experiments and experimental settings. Section 6 provides results and comparisons. Finally, Section 7 provides a discussion of the proposed research, findings, and limitations. Section 8 presents conclusion and some future work.

## 2. Related Work

Currently computer vision techniques using deep learning are being used specifically for categorizing medical and natural images [3,22]. As a direct result of this endeavor’s success, many academics are presently using deep convolutional neural networks (DCNNs) to diagnose thoracic illnesses based on chest radiographs. However, because the vast majority of these DCNN models were designed to address a variety of problems, they typically have three shortcomings: (1) they frequently fail to take into account the characteristics of various types of thoracic diseases; (2) they frequently make the wrong diagnosis because they do not focus solely on aberrant areas; and (3) their diagnosis can be challenging to understand, which limits their utility in clinical practice.

Traditional clinical experiences, according to [23], have demonstrated the value of lesion site attention for better diagnosis. The authors in [23] developed disease location attention-guided network (LLAGnet) that focuses on lesion site features that are discriminative in chest X-rays for thoracic disease categorization using multiple labels. The authors in [24] utilized a transfer learning technique for deep learning convolution layer training and worked on a small segment of data. The researchers in [25] worked on a small dataset of a few hundred images for testing and training. They used a neutrosophic approach by applying various deep convolutional models for COVID-19 classification from different lung infections. They utilized a small amount of data, which caused overfitting. The paper [26] utilized the heuristic optimization algorithm ROFA for the classification and segmentation of pneumonia by applying different thresholds. The drawback of their approach is that the small number of pixels taken for analysis do not determine the correct location for segmentation, resulting in low accuracy for segmentation. In the research of [27], the authors utilized VGG-SegNet for pulmonary nodule classification and Lung-PET-CT-Dx for their segmentation. However, due to various parameters, the model does not tackle them and has low accuracy. The researchers in [28] proposed the CheX-Net CNN model, which is the most current and sophisticated one. CheX-Net takes in chest X-ray images and generates a heatmap that shows the locations of the regions most likely to be affected by the disease. Regarding recognizing pneumonia in 420 images of X-ray, CheX-Net outperformed four experienced radiologists on average. On the other hand, the paper’s [28] proposed model is a DenseNet variation that has not undergone any significant alterations, with the purpose of learning representations with little to no monitoring. The network weights were trained using images from ImageNet.

The research in [20] recommended employing Consult-Net to develop relevant feature representations that might be used for the classification of lung illnesses. The Consult-Net project’s goal is to overcome the obstacles posed by a broad set of diseases and by the influence of irrelevant areas in the chest X-ray classification. Study primarily focuses on classifying thoracic disorders shown in chest X-ray. Authors in [20] presented two-branch architecture known as Consult-Net for learning discriminative features to achieve two goals simultaneously, as Consult-Net is made up of two distinct parts. A feature selector bound by an information bottleneck retrieves important disease-specific features based on their relevance in the first step of the procedure. Second, using a feature integrator based on spatial and channel encoding, the latent semantic linkages in the feature space were improved. Consult-Net integrates these unique characteristics to increase the accuracy of thoracic illness categorization in CXRs.

A DCNN (Thorax-Net) is proposed in [14]. It aims to utilize chest radiography to diagnose 14 thoracic diseases. Thorax-Net features both an attention and a categorization branch. The classification branch created feature maps and the attention branch can capitalize on the relationship between class labels and clinical concerns. A diagnosis can be formed by integrating the data from two components, averaging and binarizing them, then feeding the results into the trained Thorax-Net and a chest radiograph. When trained with internal data, Thorax-Net outperforms other deep models, with AUC ranging from 0.7876 to 0.896 in each trial.

Due to the absence of substantial annotations and abnormalities in pathology, CAD diagnosis of thoracic disorders is still complex. To address this CAD challenge, the paper [29] proposed a model known as the triple-attention learning (A3 Net) system. By merging three independent attention modules into a single, coherent framework, the proposed model unifies the processes of learning attention scale-wise, channel-wise, and element-wise. The feature extraction backbone network is a pre-trained version of the DenseNet-121 network. The deep model is explicitly encouraged to focus more on the feature maps’ discriminative channels.

The initial stage in developing automated radiology classification is identifying relevant diagnostic disease in X-ray. These elements can assist in making a diagnosis. The problem is that these properties are highly non-linear, making definition difficult and providing an opportunity for subjectivity. The model must be trained to apply a sophisticated non-linear function, mapping an image with feature, to extract these features (fimg). Previous authors created a DCNN for extracting these complex non-linear features [30].

Deep CNN has a few disadvantages, the most significant being the difficulties connected with vanishing gradients and the massive number of parameters required [30]. Training deep networks for medical applications is difficult due to a lack of medical datasets (this dataset contains just about 3999 patient records). Furthermore, it is known that network is quite sensitive at beginning when it is trained from the scratch. This was established in the paper [31], which demonstrated that vanishing gradients occur in an unstable training process when a deep neural network is not properly initialized, causing the training process to be unstable. To identify qualities that are complementary to one another, Chen et al. [4] introduced DualCheXNet, a dual asymmetric feature learning network. However, the algorithms currently in use for categorizing CXR images do not take any knowledge-based information into account [4]. Instead, the emphasis is on the development of useful representations using a range of deep models. Furthermore, as the network grows in size, issues with vanishing gradients may appear on individual CXR images.

Iterative attention-guided curriculum learning was used in [32] to enhance localization performance under weak supervision and thoracic disease categorization. The results are likely affected by the attention-aware networks’ continued inability to identify goal regions accurately. This is due to a lack of expert-level supervision or instruction.

Similarly, their performance suffers greatly from imbalance data when the number of parameters increases and the existing model cannot handle large parameters, which causes incorrect classifications. On the other hand, concerns about overfitting and vanishing gradients have developed as the model’s depth has increased [20]. Furthermore, a single network with a greater depth of model is more likely to miss crucial distinguishing intermediary layer features. These characteristics are frequently subtle, yet they are critical for identifying hard-classified anomalies in CXRs. These difficulties have evolved into bottlenecks, impeding the deep extension of the ImageNet [4] model. Although 2D LSTM uses parameters more than 2D RNN. It is less efficient at runtime than 2D RNN for large images due to the issue of exploding or vanishing gradients. It is possible to overfit, especially when there are training data [33].

Gradient vanishing issue is detected in training phase as the network is fine-tuned using pre-trained models. It is likely that the lower layers of the network will not be well-customized, since gradient magnitudes (back-propagated from the training mistake) swiftly decline. Conceptual appearance of the disease is detected in top layers. The author suggested training of network one layer at a time and building from scratch the P-Net to avoid this issue [33].

The difficulty of appropriately identifying images is exacerbated due to high degree of similarities between distinct classes and the scarcity of data for specific conditions. Because the situations visually resemble one another, CXR images do not adequately reflect the entire spectrum. This is especially true for those who have two or more disorders. When CNNs are trained with many parameters, this proximity may result in overfitting, even for categories with small samples. Among the nearly 100,000 total images in the ChestX-ray14 collection, the “Hernia” positive detected are only 227 [22].

Existing models have several flaws, such as vanishing gradients as network size increases, network parameter optimization [4,34], and overfitting when a patient has many illnesses (the model becomes confused when identifying the condition using small data). Another shortcoming of method is that it does not fully address the issue of class imbalance (some diseases may have more images than others) [19]. As a result, the present model cannot train to the same level of accuracy for each of the 14 diseases, resulting in inaccurate disease detection. In addition, existing models do not tackle the correlation between different diseases [14,20]. Different models related to DCNN are being utilized for identification of thoracic diseases. There are issues related to model training, datasets, and proper labeling of the thoracic diseases. Increasing the relevant dataset sizes, obtaining more accurate labeling from professionals, and using a proper training dataset will help the DCNN models perform better.

## 3. Dataset

The dataset, its preprocessing and class imbalance issues are discussed in this section.

### 3.1. ChestX-ray14

ChestX-ray14, a hospital dataset consists of 112,120 chest radiographs of 30,805 participants as frontal-view [1]. There are 14 illness image labels on these chest radiographs, including cardiomegaly, atelectasis, mass, infiltration, effusion, pneumothorax, nodule, pneumonia, edema, consolidation, fibrosis, emphysema, and pleural thickening. These images were initially stored in PNG format before being scaled to 1024 × 1024 [1]. There are one to many labels of illness in the remaining 51,708 images that are retrieved from linked radiology reports by NLP. No sickness is found in 60,412 images (they are typical instances), whereas the remaining 51,412 images have one to many labels of diseases. It is expected that these labels are more than 90% accurate. The data is split into 14 distinct thorax disorders. The dataset was formally split at the individual patient level into a training subset consisting of 70% of the participants and a testing subset consisting of 20% of the patients and 10% for validation. Same patient will be found only in either training or testing subcategories. The testing set has 15,684 samples having one to many labels and 9912 are unlabeled, whereas training set contains 36,024 samples having one to many labels and 50,500 are unlabeled [1,14].

### 3.2. Preprocessing

As compared to the ImageNet dataset for classification purposes, the ChestX-ray14 dataset has very few spatial patterns of diseases included in images of 1024 × 1024 pixels, which creates issues for deep learning models development and computing hardware. ChestX-ray14 consists of 112,120 X-rays from 30,805 subjects with 14 diseases including cardiomegaly, atelectasis, consolidation, infiltration, effusion, fibrosis, mass, edema, pneumonia, emphysema, nodules, pneumothorax, hernia and pleural thickening. There are 60,412 images in the dataset, with no illness labeled as a normal instance, and 51,708 images in the dataset with multiple disease classifications. More than half of the photos in the sample are normal cases. Thus, a technique is used to encode each class label to 14-dimensional vector where a “1” denotes an instance relevant to thoracic disease and a “0” signifies the absence of sickness. The preprocessing of chest X-ray images occurs in two steps. The model resized the input X-rays from 1024 × 1024 to 224 × 224 pixels. Then, it used the weighted cross-entropy loss technique on the data samples to reduce the class imbalance problem while avoiding overfitting by using the ReLU layer as the activation function per convolution layer. Finally, by employing the ReLU layer as the activation function, the model applied the weighted cross entropy loss strategy to handle overfitting problem.

### 3.3. Class Imbalance

ChestX-ray14 dataset is significantly biased. Around 8000 X-ray images are available for training in the categories of atelectasis, cardiomegaly, and effusion, but only approximately 2000 images are available for edema, emphysema, and hernia. The most popular ways to deal with class imbalance are oversampling training data, undersampling training data, and penalizing classification. By including a positive/negative balancing factor in the multi-label classification loss layer eliminates the overfitting problem. There are more zeroes than ones in our one-hot-like image labeling that cause overfitting; by adding a balancing factor to the multi-label loss layer, we improve the accuracy of the proposed model. As a result, we used the well-known weighted cross-entropy loss to handle classification for increasing the model’s penalty for improperly classifying the minority group while it was being trained. Weighted cross-entropy loss was found to assist in reducing class imbalance by enhancing AUC per-class and its average across various diseases.

## 4. Proposed Z-Net Model

**Figure 1:**
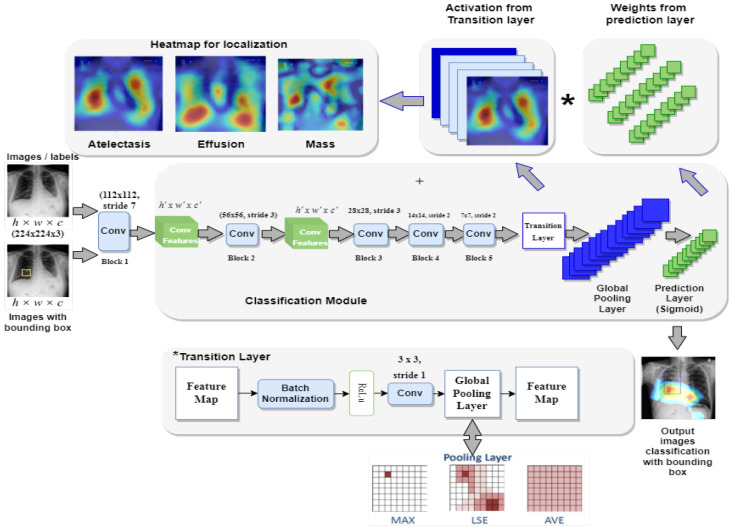
Framework of the proposed Z-Net model.

This section presents the proposed framework Z-Net for ChestX-ray14 classification and localization. Figure 1 depicts overall method that mainly consists of convolution layer, transition layer, global pooling layer, and prediction layer.

### 4.1. Z-Net Framework

The main objective of the Z-Net is evaluation of the diseases existence in each X-ray image and then use activation function and network weights to identify diseases. To address this issue, a technique using several classification labels is used. The proposed model’s architecture is depicted in Figure 1. DenseNet-121 was employed as the model’s foundation, as it had been used in earlier established approaches for unsupervised object detection and localization. The fully connected and final classification layers from the model’s classification module were deleted to perform network surgery on the pre-trained DenseNet-121 model shown in the Figure 1. Instead, toward the end of the process, the suggested model employs a global pooling layer, transition layer, prediction layer and loss layer (after the last convolution layer). The Z-Net based on DenseNet-121 takes the weights from the pretrained DenseNet-121. Only transition layer and the prediction layer are trained since beginning. The weights of spatial map from the prediction inner-product layer, which are weight-based learning features, were combined with the activation from the transition layer, which is collection of spatial image properties, to identify the precise location of the disease.

### 4.2. Components of the Z-Net

Here we explain each component of the proposed model. There are five components of Z-Net, which are presented in the following subsections:

- Multi-labeling;
- Transition Layer;
- Loss Layer;
- Global Pooling and Prediction Layer;
- Bounding Box Generation.

Pseudocode for the proposed Z-Net model is shown in Algorithm 1.   
**Algorithm 1:** Pseudocode of Z-Net model
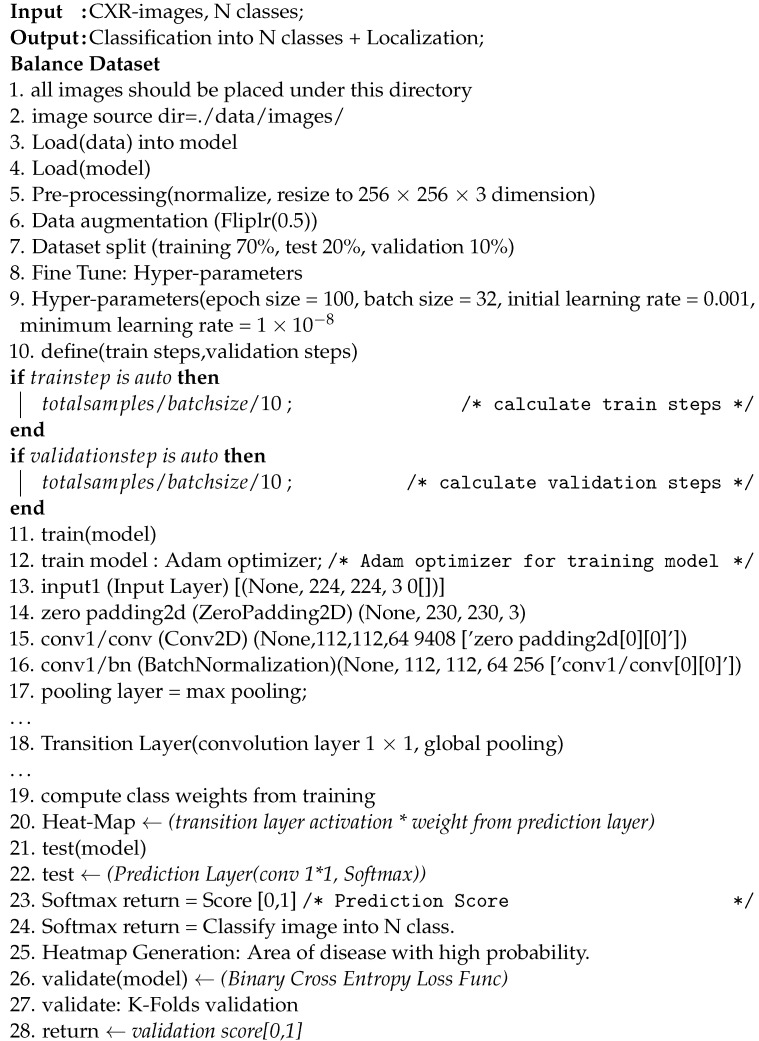


#### 4.2.1. Multi-labeling

In addition to a wide range of image-label representation options, a multi-label classification loss function is provided. The system employs a 14-dimensional vector for each image in which a 1 shows disease presence in the X-ray image, whereas a complete absence is shown as a zero vector that indicates the absence of disease in any of the 14 categories. In the multi-label classification, jobs are converted into loss settings comparable to those produced by regression using this labeling technique.

#### 4.2.2. Transition Layer

For most models, a transition layer is necessary to convert activations from earlier layers into an output dimension that is consistently 8, 16, or 32 in S × S × D. This is due to the large number of various pre-trained model designs that are used. The proposed model utilized a transition layer for training purposes on the large dataset. The variable D denotes the spatial feature location dimension (i,j), (i,j)e 1, …, S, which varies based on the model and the conditions; for example, D = 224 is used for the Z-Net model. In order to use the activations of this layer further to produce the heatmap during the pathological localization stage, the transition layer must reliably send down the weights from previously trained Z-Net models.

#### 4.2.3. Loss Layer

Binary cross-entropy loss functions are included in the model, enabling numerous labels to be used to validate it. The loss rate for the proposed model is computed using binary cross-entropy. The image labels utilized in the experimental study are limited, indicating that there are many more zeroes than ones, and learning of positive scenarios is difficult for the model (images with pathologies). This is due to the uneven distribution of the pathological and “Normal” classifications and to our image identification technique, which is equivalent to a one-hot. As a result, we included the negative and positive balancing components P and N to assist in learning from valuable instances. As an illustration, the weighted CEL, sometimes referred to as the W-CEL, is described in Equation (1).

(1) $$\beta_{P}\sum_{y_{c}=1}-\ln\left(f\left(x_{c}\right)\right)+\beta_{N}\sum_{y_{c}=0}-\ln\left(1-\left(f\left(x_{c}\right)\right)\right)$$

#### 4.2.4. Global Pooling and Prediction Layer

The proposed Z-Net model classification network uses prediction and pooling layers of the multi-label image classification framework to create probability maps for diseases. These probability maps are also known as heatmaps. The highest point on a heatmap almost invariably corresponds to the location with the highest likelihood of displaying a disease pattern. After the transition layer is complete, a global pooling operation is performed using the prediction layer’s weights for the spatial map. The activation from the transition layer (S × S × D) is multiplied with prediction layer weights (D × C), which results in class-wise weighted spatial activation in the proposed model (S × S × C).

Pooling layer is in charge for deciding which data to pass on to the system below it. The model will employ the LogSumExp (LSE) pooling approach, one of the available pooling methods, to accomplish this task.

#### 4.2.5. Bounding Box Generation

Each of these bounding boxes represents a different subtype of thoracic disease. This is accomplished by multi-label classification by predicting the geographical location of the objects. The heat-intensity map creates the sense of a strong link between illness and disease. The model uses the color combination [0, 255] for temperature normalization, and the threshold value was set at [60, 180]. Using this strategy, we are able to target affected areas more precisely.

### 4.3. Disease Localization

In multi-label disease classification, for each pathology candidate, the proposed model generates the bounding boxes utilizing transition layer activation and prediction layer weights. For multi-label disease classification, this model not only creates bounding boxes for each pathology but also produces illness heatmaps. The heatmap represents the disease area in red, with highest probability of disease being in that area.

## 5. Experimental Setup

Here we discuss the dataset preparation and experimental settings.

### 5.1. Dataset Preparation

**Figure 2:**
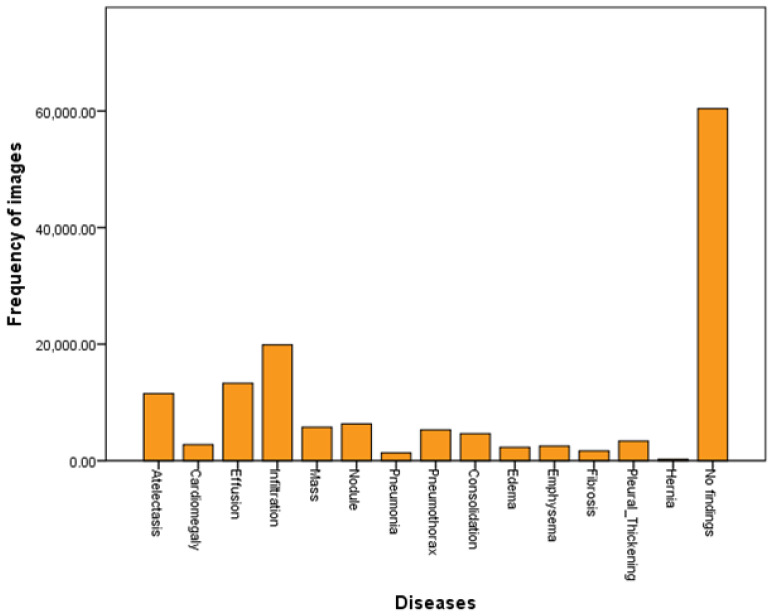
Dataset visualization.

The Z-Net model is analyzed and validated using the benchmark ChestX-ray14 dataset as shown in the Figure 2. The dataset consists of 112,120 images from frontal viewpoint, with 51,708 images including at least one illness. There are 60,412 images that are normal as shown in the Table 1 and visualized in Figure 2. We randomized dataset and divided it into three subgroups with the aim of fine-tuning the CNN using stochastic gradient descent (SGD): training (70%), testing (20%), and validation (10%), as shown in Table 2. We reported the outcomes of eight thoracic diseases in the testing set. Moreover, 983 cases having 1600 various disease B-Boxes labeled on them are the only ones utilized for ground truth in the testing to establish how precisely an illness is localized (not for training purposes).

### 5.2. Experimental Settings

In this research work, the ChestX-ray14 dataset [1] is utilized, that comprises 112,120 X-ray images of 30,805 distinct patients having 14 different types of ailments (images may have multiple labels). The dataset was obtained from the NIH Clinical Center’s clinical PACS database. It is now the most comprehensive chest X-ray open dataset. These images were initially distributed in PNG format at 1024 × 1024 pixels, but they have been re-scaled to 256 × 256 pixels. We utilized the data splits at identical patient-level used in official dataset [1]. Around 70% of the images in official dataset are used for training, 10% for validation, and 20% for testing.

Z-Net model used data augmentation, during training phase, to boost the overall data and reduce overfitting. The model starts by reducing the image resolution to 256 × 256 pixels. The suggested Z-Net model cropped the image to 224 × 224 pixels and randomly translate image between range −12 pixels to 12 pixels. After that image is normalized using standard deviation and average of training data. Weights from the first convolutional layer were used to initialize the proposed model. Meanwhile, the convolutional layer extracted image features and created a feature map. This feature map was then transferred to the next convolutional layer for additional learning. The Adam optimizer and the SGD network were used to optimize the recommended model, with a weight decay of 0.0001 and momentum of 0.9. As a result, batch size is 32. After 20 epochs, learning rate is divided by 10, and the process is started at a learning rate of 0.001, as seen in Table 3.

## 6. Results and Comparison

In this section, we describe results achieved by the Z-Net model.

### 6.1. Loss Function

**Figure 3:**
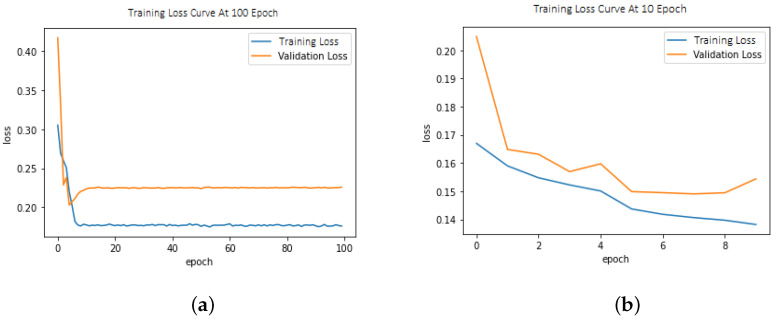
(**a**) Training/validation loss curve at epoch size = 100; (**b**) Training/validation loss curve at epoch size = 10.

This model demonstrated improved performance using balanced loss functions as shown in Equation (1). The weighted cross-entropy loss (W-CEL) performs better than CEL. Validation loss due to CEL is shown in Figure 3a,b.

### 6.2. Multi-Label Disease Classification

**Figure 4:**
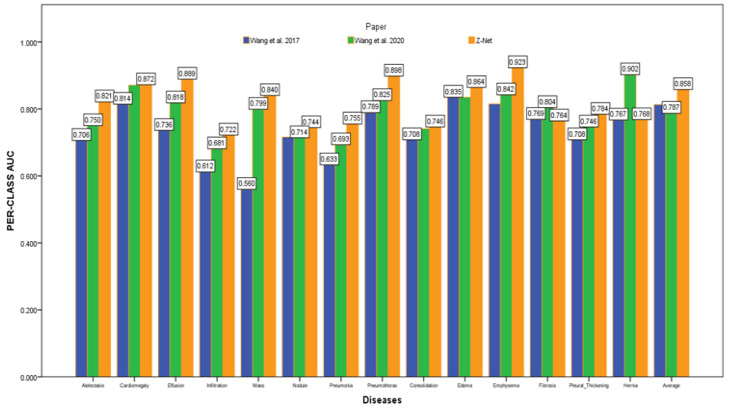
AUC comparison with other models.

After initializing the Z-Net framework with DenseNet-121 before network surgery, Figure 4 displays multi-label classification based AUC-ROC curves for 14 classes of disease. AUC value comparisons are shown in the Table 4. The proposed model achieves the best quantitative performance across many models. Comparatively well-recognized classifications are “Cardiomegaly” AUC = 0.8725 and “Pneumothorax” AUC = 0.8980. The likelihood of detecting diseases with minute symptoms, such as “Mass” (AUC = 0.8406) and “Nodule,” might be significantly lower. Because there are so many possible forms of mass, it is notoriously hard to define it. Less than 1% of X-rays were identified as infiltration and hernia, which is likely the cause of the poor performance of “Infiltration” (AUC = 0.722) in our patient sample. This discovery agrees with the outcomes of a comparison of the object identification capabilities of various cutting-edge research papers.

### 6.3. Evaluation Metrics

AUC Score: The proposed Z-Net model used identical experimental inputs as the baseline models investigated in this study. The proposed method does not need prior training, in contrast to DCNN. An indication of performance used to evaluate imbalanced learning is AUC value. This number is utilized by the proposed Z-Net model to gauge how well each of the 14 illness categories is doing. Y-axis of the ROC indicates true positive values, while x-axis indicates false positive. ROC curve depicts the relative benefits and costs of identifying genuine positives instead of false positives. AUC measures the chance that the model would assign more value to a a positive case at random instead of negative. It ranges from 0.5 to 1. If AUC = 1, the classifier properly separates the classes. If the AUC value is greater than 0.5 and less than 1, the classifier has a decent chance of distinguishing between the various classes. If AUC value is 0.5, classifier is not effectively classifying positive and negative. Therefore, higher AUC value denotes a more accurate classifier.

The ROC graph evaluates the classification performance of the model for various threshold values. AUC is a metric that assesses a classifier’s ability to distinguish between a number of distinct classes, which is 1−Specifity. Specifity is defined as:

(2) $$Specificity=\frac{TN}{FP+TN}.$$

In Equation (2), *TN* or true negative means correct prediction counts of the negative class whereas *FP* or false positive means incorrect prediction counts of the positive class.

### 6.4. Comparative Analysis

**Figure 5:**
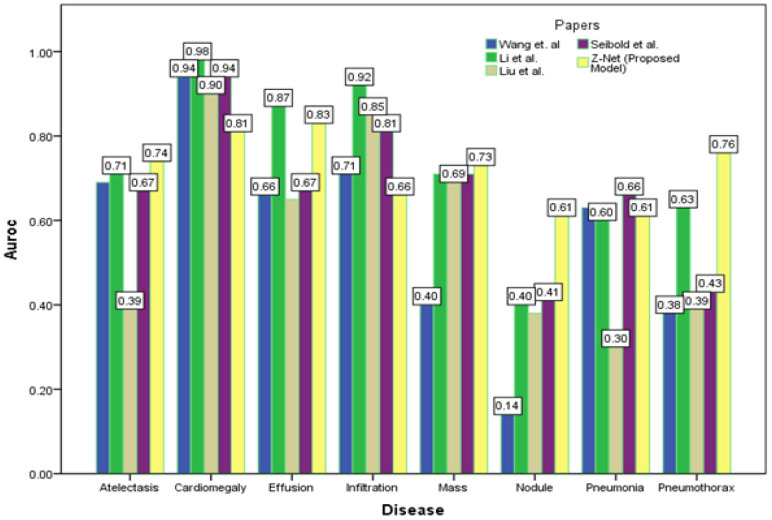
Comparison of localization results with latest research.

Figure 4 depicts the multi-label classification of 14 diseases using the proposed approach, and Table 4 contains the related AUC values. The quantitative analysis depicts that Z-Net achieved best results in comparison with other models. The “Atelectasis” (AUC = 0.82), “Infiltration” (AUC = 0.72), “Pneumothorax” (AUC = 0.89), “Effusion” (AUC = 0.88), and “Pneumonia” (AUC = 0.75) are best classified by the proposed approach when compared to other diseases. “Hernia” has a very low performance (AUC = 0.76 as compared to others) due to a lack of disease cases in our sample population. Finally, we generated a heatmap and bounding box for the diseases by utilizing transition layer activation and weights from the predicted layer. The generated bounding boxes (B-Boxes) are compared with ground truth (GT) boxes present in dataset. The standard intersection-over-union ratio (IoU) is utilized for calculating accuracy of generated B-Boxes against the ground truth boxes. The localization results of the proposed approach are improved when T(IoU) = 0.1 in “Atelectasis” = 0.743, “Mass” = 0.733, “Nodule” = 0.612, and “Pneumothorax” = 0.762. The low accuracy in the cases “Cardiomegaly” and “Infiltration” is due to fewer B-Boxes in comparison with the dataset in the sample population. Table 5 demonstrates the localization accuracy of computerized bounding boxes against ground truth values, as shown in Figure 5.

### 6.5. CAM Visualization

**Figure 6:**
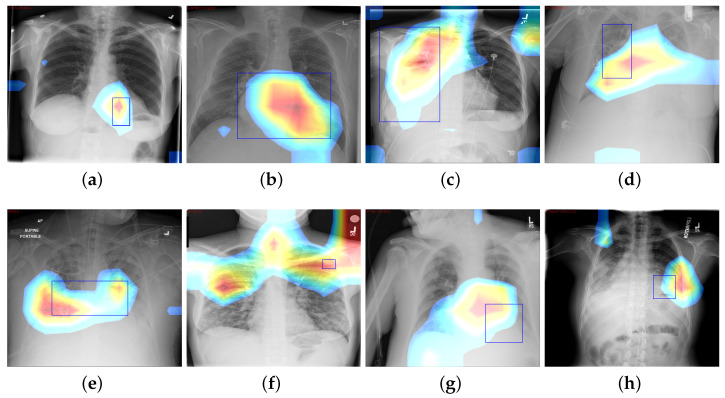
CAM visualization results by the Z-Net model with bounding boxes around infected areas of the diseases: (**a**) Atelectasis, (**b**) Cardiomegaly, (**c**) Effusion, (**d**) Infiltration, (**e**) Mass, (**f**) Nodule, (**g**) Pneumonia, and (**h**) Pneumothorax.

We build disease heatmaps for each pathology by obtaining activation values from final convolution layer. Feature map $M_{c}$ (for the most prominent features) is generated by adding related weights, as shown in Equation (3):

(3) $$M_{c}=\sum_{k=1}^{n}W_{c}{,}_{k}F_{k}$$

where $W_{c}{,}_{k}$ is value of final convolution layer at feature map *k* toward the pathology *c*, whereas $F_{k}$ is the *k*th feature map. We use CAM to localize diseases by emphasizing the diseased areas of images relevant for accomplishing classification of some illness. In spite of the modest amount of 984 annotated bounding boxes in comparison with overall data, it is adequate to provide a reasonable assessment of our proposed framework’s illness localization performance. Figure 6 depicts different instances of CAM visualization.

## 7. Discussion

The fundamental contribution of this study is the development of the Z-Net model and its integration with global characteristics. The integration was carried out to identify areas of pathological anomaly and allow categorization to focus on such illness zones. Given that the dataset consists of 983 bounding boxes, it was critical to verify that heatmaps created using class labels of images and ground truth bounding boxes match one another. The model was given learning heatmaps for a normal case as well as eight cases with the following conditions, in listed order: atelectasis, cardiomegaly, effusion, infiltration, mass, nodule, pneumonia, and pneumothorax. For each occurrence, the heatmap, which illustrates areas of high activation in the red color whereas areas of low activation in the blue color. Illness boundary boxes are overlaid on the image. This shows that the learned heatmaps fit the bounding boxes fairly well, even when the size varies. Fortunately, these areas of elevated activity were found to be outside the heart and lungs. Ability of Z-Net in detecting pathological anomalies in the vast majority of chest radiographs explains the performance improvement that our suggested model achieves. Limitations of the study include imbalances in the dataset and the size of the data, which may affect training and performance. Also, due to small number of annotations (as it was only supervised by image-level class labels), there were inadequate clinical abnormality annotations. In addition, there is a need to have labels that provide relationships among various thoracic diseases.

## 8. Conclusions and Future Work

This research proposed a unified DL-based Z-Net framework for performing classification and localization of thoracic disease on chest X-rays images using patient-wise split of the ChestX-ray14 data. The Z-Net framework makes use of DenseNet-121 as its backbone, which is a form of learning that is only weakly supervised. The proposed framework overcomes the issue of class imbalance in the ChestX-ray14 dataset by using a technique in the proposed model called weighted cross-entropy loss (W-CEL), that penalizes the classification by adding cost during training on minority classes for classification purposes. The usage of noisy multi-class illness labels is incorporated into this system. In this research, we have described the benefits of a network that was proposed to use transfer learning to learn the disease-specific features through the use of convolutional layers, as well as the global feature recalibration that was accomplished through the use of global pooling layer blocks that were placed in between dense blocks. Heatmaps, which are a byproduct of obtaining information from the activation of the transition layer and the weight of the prediction layer, identify the infected area of disease with high probability and are used to demonstrate the symbolic strength of the recommended network. In addition to that, this demonstrates that the proposed model may be interpreted in several ways. The proposed approach achieved the highest mean AUC score of 85.8% as compared to existing studies. In conclusion, both quantitative as well as qualitative studies show that our framework outperformed latest research models.

In future, we aim to develop a more accurate and sophisticated model that can easily locate the small intrinsic and overlapping elements of disease in the X-ray image and that can develop the correlation between different diseases.

## Figures and Tables

**Table 1:** Dataset details.

Disease	Frequency of Images
Infiltration	19,871
Atelectasis	11,535
Pneumothorax	5298
Consolidation	4667
Edema	2303
Fibrosis	1686
Emphysema	2516
Effusion	13,307
Pleural Thickening	3385
Pneumonia	1353
Cardiomegaly	2772
Mass	5746
Nodule	6323
Hernia	227
No Finding	60,412

**Table 2:** Dataset split ratio.

Train	Test	Validation
70%	20%	10%

**Table 3:** The hyper-parameter values of proposed model Z-Net.

Parameters	Value
Number of epochs	100
Optimizer	Adam
Batch size	32
Initial learning rate	0.001
Minimum learning rate	$1\times 10^{-8}$
Activation function	Sigmoid
Loss function	Binary Cross-Entropy Loss

**Table 4:** Comparison of AUC scores of our proposed Z-Net technique with latest research.

Pathology	Wang et at. [1]	Li et al. [3]	CheXNet [28]	Thorax-Net [14]	Guan et al. [20]	Seibold et al. [21]	Proposed Z-Net Model
Atelectasis	0.706	0.800	0.779	0.750	0.785	0.78	0.821
Consolidation	0.708	0.800	0.754	0.741	0.763	0.75	0.746
Infiltration	0.6128	0.700	0.689	0.681	0.699	0.71	0.722
Pneumothorax	0.789	0.870	0.851	0.825	0.871	0.81	0.898
Edema	0.835	0.880	0.849	0.835	0.850	0.86	0.864
Emphysema	0.815	0.910	0.924	0.842	0.924	0.95	0.923
Fibrosis	0.769	0.780	0.821	0.804	0.831	0.85	0.764
Effusion	0.736	0.870	0.826	0.818	0.835	0.84	0.889
Pneumonia	0.633	0.670	0.735	0.693	0.738	0.74	0.755
Pleural Thickening	0.708	0.790	0.792	0.776	0.746	0.90	0.784
Cardiomegaly	0.814	0.870	0.881	0.871	0.899	0.88	0.872
Nodule	0.716	0.750	0.781	0.714	0.775	0.81	0.744
Mass	0.560	0.830	0.830	0.799	0.838	0.84	0.840
Hernia	0.767	0.770	0.932	0.902	0.922	0.94	0.768
Mean AUC	0.813	0.806	0.818	0.787	0.822	0.83	0.858

**Table 5:** Comparison of localization results with the latest papers.

Disease	Wang et al. [1]	Li et al. [3]	Liu et al. [35]	Seibold et al. [21]	Z-Net (Proposed Model)
Atelectasis	0.69	0.71	0.39	0.67	0.74
Cardiomegaly	0.94	0.98	0.90	0.94	0.81
Effusion	0.66	0.87	0.65	0.67	0.83
Infiltration	0.71	0.92	0.85	0.81	0.66
Mass	0.40	0.71	0.69	0.71	0.73
Nodule	0.14	0.40	0.38	0.41	0.61
Pneumonia	0.63	0.60	0.30	0.66	0.61
Pneumothorax	0.38	0.63	0.39	0.43	0.76
Mean	0.57	0.73	0.60	0.66	0.71

## Data Availability

Dataset used in this paper is publicly available at https://nihcc.app.box.com/v/ChestXray-NIHCC, accessed on 21 October 2023.
